# Measuring the Electronic Bandgap of Carbon Nanotube Networks in Non-Ideal *p-n* Diodes

**DOI:** 10.3390/ma17153676

**Published:** 2024-07-25

**Authors:** Gideon Oyibo, Thomas Barrett, Sharadh Jois, Jeffrey L. Blackburn, Ji Ung Lee

**Affiliations:** 1 College of Nanotechnology, Science, and Engineering, State University of New York-Albany, Albany, NY 12203, USA; oyibog@sunypoly.edu (G.O.); barrettl@sunypoly.edu (T.B.); joiss@sunypoly.edu (S.J.); 2 National Renewable Energy Laboratory, Golden, CO 80401, USA; jeffrey.blackburn@nrel.gov

**Keywords:** carbon nanotube, *p-n* diode, bandgap, excitons, binding energy

## Abstract

The measurement of the electronic bandgap and exciton binding energy in quasi-one-dimensional materials such as carbon nanotubes is challenging due to many-body effects and strong electron–electron interactions. Unlike bulk semiconductors, where the electronic bandgap is well known, the optical resonance in low-dimensional semiconductors is dominated by excitons, making their electronic bandgap more difficult to measure. In this work, we measure the electronic bandgap of networks of polymer-wrapped semiconducting single-walled carbon nanotubes (s-SWCNTs) using non-ideal *p-n* diodes. We show that our s-SWCNT networks have a short minority carrier lifetime due to the presence of interface trap states, making the diodes non-ideal. We use the generation and recombination leakage currents from these non-ideal diodes to measure the electronic bandgap and excitonic levels of different polymer-wrapped s-SWCNTs with varying diameters: arc discharge (~1.55 nm), (7,5) (0.83 nm), and (6,5) (0.76 nm). Our values are consistent with theoretical predictions, providing insight into the fundamental properties of networks of s-SWCNTs. The techniques outlined here demonstrate a robust strategy that can be applied to measuring the electronic bandgaps and exciton binding energies of a broad variety of nanoscale and quantum-confined semiconductors, including the most modern nanoscale transistors that rely on nanowire geometries.

## 1. Introduction

The ‘electronic bandgap’ of a semiconductor is defined as the energy difference between the lowest-energy single-particle electron and hole levels. This fundamental property defines many technologically critical properties and processes of a semiconductor, including exciton binding energies, reduction and oxidation potentials in (photo)catalytic reactions, and achievable ranges for quasi-Fermi level splitting in solar cells. In transistors and diodes, the bandgap can determine the leakage current of these devices, an important metric that can determine the efficiencies of the systems they enable. Measuring the electronic bandgap, also known as the transport bandgap, of semiconducting single-walled carbon nanotubes (s-SWCNTs), is challenging. Traditional techniques such as optical absorption do not work because of the weak oscillator strength of band-to-band transitions in quasi-one-dimensional materials. Instead, the optical absorption is dominated by intense excitonic transitions that arise from the strong coulomb binding between electrons and holes [1]. The use of scanning tunneling spectroscopy poses other problems due to screening from the metal substrates used in these measurements [2]. Here, we measure the electronic bandgap of polymer-wrapped s-SWNTs by creating *p-n* diodes with an ideality factor of 2, one of only two types of diodes that allows the measurement of the bandgap through transport properties. The technique we show and the conclusions we draw are broadly applicable. For example, we note that while s-SWNTs may represent the extreme limit of one-dimensional confinement, the same challenges in measuring the bandgap in s-SWNTs will also apply to current and future transistors as they are shaped into nanowire geometries to allow continued scaling [3,4].

The use of polymer wrapping in the purification and sorting of s-SWCNTs has given unprecedented access to highly homogeneous chiralities with varying diameters and bandgaps [5,6]. Polymer-wrapped s-SWCNTs are solution-processable and are already being used in the fabrication of carbon nanotube microprocessors [7,8], solar cells [9,10,11,12,13], thermoelectrics [14,15], and light emitting devices [16,17]. Despite their potential for widespread use, there remain gaps in our understanding of their fundamental properties. While charge transport in polymer-wrapped s-SWCNT networks is already an established field [18], their intrinsic transport bandgap has remained elusive despite its fundamental and technological importance. Here, we provide measurements of the electronic bandgap of polymer-wrapped s-SWCNTs.

The *p-n* diode is one of the most fundamental building blocks of optoelectronic and electronic devices and it can also be used to study the bandgaps of semiconductors [19,20,21]. It is already well known that the optical transitions of carbon nanotubes are dominated by excitons (electron–hole pairs bound by a binding energy, *E_b_*) [1]. The optical bandgap, which is the first excitonic transition, *E*_11_, of s-SWCNTs is therefore smaller than the electronic bandgap *E_g_*, where *E_g_ = E*_11_ *+ E_b_*. In this work, we fabricate *p-n* diodes using networks of polymer-wrapped s-SWCNTs that allow us to measure the diameter-dependent s-SWCNT electronic bandgaps (*E_g_*) and binding energies (*E_b_*). Our results are consistent with theoretical and experimental values from previous works on the intrinsic bandgap of s-SWNTs but renormalized by the dielectric environment [2,22,23].

## 2. Device Fabrication and Results

**Figure 1:**
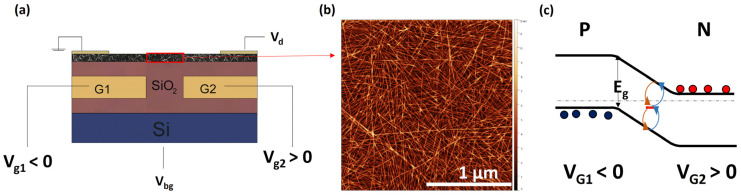
(**a**) Device architecture showing *p-n* diode formed using buried split gates G1 and G2 to electrostatically dope the s-SWCNT network. The back gate V_bg_ is used to ensure ambipolar conduction by measuring the transfer curves (see Supplementary Figure S2). (**b**) Typical AFM image of s-SWCNT network used in forming *p-n* diodes. (**c**) Band diagram of *p-n* diode showing mid-gap states in the intrinsic region dominating the leakage current (*I_o_*) due to generation and recombination of carriers. We use the temperature dependence of *I_o_* to calculate the bandgap of the s-SWNT network.

We fabricated *p-n* diodes using electrostatic gating techniques, described in our previous works [11,24,25,26], as shown in Figure 1 (see also Figure S1 for a device schematic with a full description of dimensions). Using buried split gates G1 and G2 with split gate spacing, G, ranging from 0.1 µm to 0.5 µm, we applied opposite bias to create *p*- and *n*-doped regions on the s-SWCNT network as shown in the band diagram of Figure 1c. We note that devices with G > 0.5 µm tend to show diodes with ideality factor *n* > 2, with some having *n* > 3, which are unsuitable for this study. This behavior results from the large disorder present in these films, as we show below. Therefore, we focused on diodes with *n*~2, which allows us to extract the bandgap of different network s-SWCNTs.

**Figure 2:**
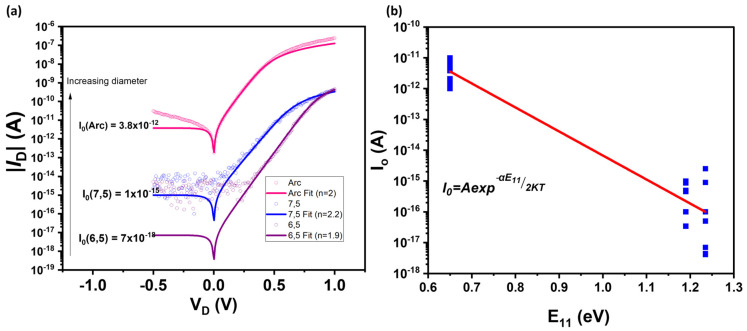
(**a**) Dark current voltage (I-V) characteristics of *p-n* diodes fabricated on networks of different s-SWCNT distributions-arc, (7,5), and (6,5)-showing leakage current (I_o_) values increasing with diameter (decreasing bandgap) and ideality factor of 2. (**b**) Leakage current (I_o_) from the compilation of representative non-ideal diodes, plotted on a natural log scale against the E_11_ optical transitions of the s-SWCNT networks. E_11_ values for our devices are obtained from the photocurrent spectra of the diodes (see Figure S3). Despite the large variation in leakage current on each s-SWCNT type, we observe an inverse dependence of the leakage current on the bandgap of the s-SWCNTs consistent with our previous results on individual nanotubes [26].

We characterized *p-n* diodes formed on polymer-wrapped nanotube networks of different diameters of largely monochiral s-SWCNTs. Large-diameter arc discharge s-SWCNTs (~1.55 nm, purchased from Carbon Solutions, Inc., Riverside, CA, USA) were extracted using PFO-BPy, whereas small-diameter (6,5) (0.76 nm) and (7,5) (0.83 nm) s-SWCNTs were extracted from CoMoCAT using PFO-BPy and PFO, respectively [5,6]. Figure 2a shows representative diode current-voltage (*I-V*) curves measured at *T* = 300 K from arc, (7,5), and (6,5) networks. We fitted the *I-V* characteristics to the diode equation $I_{D}=I_{o}\left(\exp\left(\frac{qV}{nKT}\right)-1\right)$ to extract their respective leakage currents *I_o_* and ideality factors *n*. *V* is applied voltage and *KT* is thermal energy.

For ideal diodes (*n* = 1), diffusion of minority carriers from the *p*- and *n*-doped regions dominate the reverse bias characteristics, while for non-ideal diodes (*n* = 2), generation and recombination of electron–hole pairs due to mid-gap states in the intrinsic region is responsible for the diode leakage current. We note that these are the only two types of behavior that allow one to measure the bandgap using the thermal activation energy of $I_{o}$.

In our diodes, the intrinsic region will form between the split gates. We can measure ideal behavior in nearly abrupt *p-n* diodes with intrinsic spacing of ~0.1 µm, while all devices with intrinsic spacing greater than ~0.1 µm exhibit non-ideal behavior. This trend further supports that large disorder is present in these films. Since most of the devices were made with G > 0.1 µm, we focus on these devices with *n*~2.

## 3. Generation and Recombination Leakage Current

We measured the *I-V* characteristics of non-ideal diodes at *T* = 300 K, as shown in Figure 2, with ideality factor n ~ 2, showing that the reverse bias leakage current in our devices is due to the generation and recombination from mid-gap states in the undoped intrinsic region.

We used the phenomenological Shockley–Read–Hall theory (SRH) [19] to determine the key properties of our polymer-wrapped s-SWCNT films. We assumed the films are quasi-two-dimensional and used the surface carrier generation rate developed from the SRH model. Due to the large density of CNTs we achieved, beyond the ~10 s-SWCNT/µm threshold for percolation [18,27], we treated the network as a very thin film. Using the simplest model where the trap level is at mid-gap, we obtained the surface generation rate U=ni2τ. This U gives the highest generation and recombination rate and therefore the worst estimate of the disorder density. The intrinsic carrier density is given as ni=D0e−Eg2KT. $D_{0}$ is related to the effective density of states which we have derived in our previous work for a single nanotube to be $16/3\pi dV_{pp}$, where d (0.14 nm) is the C-C length and $V_{pp}$ (2.5 eV) is the hopping energy between the nearest neighbor sites [28]. τ is the minority carrier lifetime and is inversely related to other parameters, including the trap density and the capture cross-section. The leakage current due to generation and recombination, $I_{0}=qUW$, can therefore be related to the temperature through the Arrhenius relationship, I0=Ae−Eg2KT, where A is a constant, *q* is the elementary charge, and W (0.1 μm) is the width of the intrinsic region [19]. We see that the SRH theory naturally gives Eg/2 as the activation energy, Ea.

We measured the *I-V* characteristics of more than 20 non-ideal diodes across different diameter s-SWCNT networks and plotted the leakage current (*I_o_*) values against the optical bandgap *E*_11_ of each s-SWCNT, as shown in Figure 2b. Since both the electronic bandgap, *E_g_,* and optical bandgap, *E*_11_ (see Figure S3), scale approximately inversely with diameter [23,26], they are related. We thus expect *I_o_* to depend in a similar way to the optical gap, further supporting that it is a measure of the fundamental properties of s-SWCNTs. To confirm, we used *E_g_ = αE*_11_ in I0=Ae−Eg2KT [26], where *α* is a scaling parameter. In Figure 2b, we fit to a linear slope and extract α≈0.98. We note that this value is fortuitously close to the value of 1 and that a large scatter in some of the data, as explained previously, makes the correlation only approximate. Nevertheless, the correlation in Figure 2b clearly links *I_o_* to *E*_11_ through *E_g_*.

We note that although the scatter in the data is somewhat large, we provide additional data to support the trend seen in the monochiral s-SWCNT devices by including polychiral HiPCO s-SWCNT device results in the Supplementary Information Figures S2, S3, S5, and S6. In particular, in Figure S6, one can see that the HiPCO s-SWCNT data fall outside of the monochiral trends due to the large variation in the bandgap of SWCNTs within that mixture. Within the SRH model, the large variation in the bandgap can be viewed as additional disorder that enhances the generation and recombination of minority carriers, resulting in an unusually large value for *n*, as seen in Figure S5.

**Figure 3:**
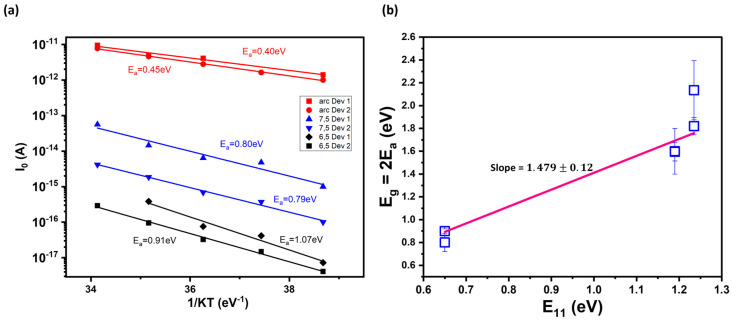
(**a**) Leakage current I_o_ is plotted on a natural logarithmic scale vs. 1/KT for two representative devices, each for arc, (7,5), and (6,5) s-SWCNTs. The slope of the linear fit is the activation energy, E_a_, according to the temperature-dependent Arrhenius relationship for I_0_. (**b**) Relationship of the measured bandgap E_g_ (2E_a_) vs. E_11_, a fundamental property of the s-SWCNT network. Each data point is the bandgap extracted from the activation energy of each device on (**a**) using the relation E_g_ = 2E_a_. The error bars account for the uncertainty in the fitting parameter for each device on (**a**).

Temperature-dependent measurements allow us to further clarify the link between the optical and electronic bandgaps. To do so, we use the SRH model for non-ideal diodes (*n* = 2) to analyze temperature-dependent measurements. Diode *I-V* curves at different temperatures (300 K–340 K) were fitted to the dark diode equation with leakage current values extracted as explained above (see Figure S4). *I_o_* was then plotted on a natural log scale vs. 1/KT and the slope fitted to obtain the activation energy according to the relationship $I_{0}\propto e^{\frac{{-E}_{a}}{KT}}$, as shown in Figure 3a. In Figure 3b, we show that the measured electronic bandgap (2E_a_) is related to the optical bandgap E_11_. Using E_11_ values from the photocurrent spectra of our devices (see Figure 3), we derived E_g_~1.48E_11_, which is close to the results from our single-nanotube *p-n* diode studies [26,28] and provides a relationship between the electronic and optical bandgaps.

Before we continue, we explain our choice to exclude devices with G > 0.5 µm and support it by extracting parameters from U. To extract parameters from U, we assume a close-packed aligned array of s-SWCNTs fills the device area and scale the leakage current accordingly in the SRH model. This scaling helps us to approximate the number of nanotubes that contribute to the generation current. As seen below, the large difference in the lifetime between our film and that of the best semiconductor interface helps to justify this approximation. Equating U to the generation rate from the leakage currents, we arrive at $\tau \sim 10^{-7}s$. In our calculation, we assume a region about 0.1 µm between the gates contributes to the generation of minority carriers. This calculation is based on AFM scans resulting in an average thickness of 2 nm, providing a linear density of 600 nanotubes/µm.

**Figure 4:**
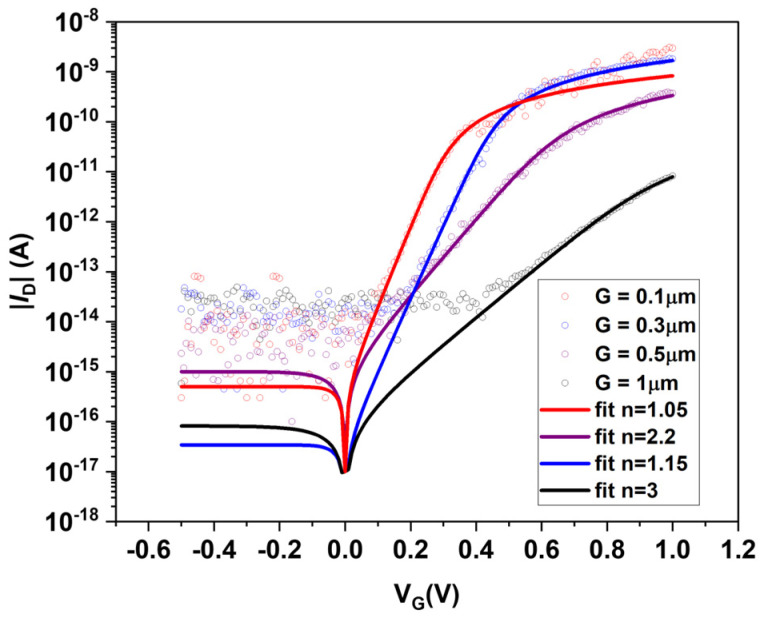
Dependence of the dark I-V characteristics on the length of the intrinsic region (G) of (7,5) s-SWCNT network *p-n* diodes. We obtain ideal diode behavior when the intrinsic spacing is 0.1 µm (nearly abrupt). As the length G increases, the diodes become non-ideal.

To further support this approximation, we show in Figure 4 the I-V characteristics of (7,5) network devices as a function of the split gate spacing. Some variation in leakage current is expected due to the variation in network thickness across devices. The most important change is the abrupt increase in the ideality factor for split gate spacing greater than 0.5 μm. This increase points to a large disorder present in our films. The minority carrier lifetime τ that we calculate is very short compared to some of the most pristine interfaces in semiconductor devices. For example, the SiO_2_/Si interface of a MOSFET is known to have minority carrier lifetimes in the order of 0.1–1 ms [29]. The short lifetime of s-SWNTs implies a large interface trap density and/or capture cross-section, and is consistent with the rapid transition to n > 2 diodes as the length of the intrinsic region increases, as shown in Figure 4. Since our analysis requires diodes with *n*~2, we chose diodes with relatively small intrinsic lengths.

Previously, we reported on large interface states that arise from the substrate on a sparse network of s-SWNTs [30]. The surface contributes an effective volume around the nanotube that can contribute to trap states. We observed that these are traps for electrons, which have a large density of states that prevented the formation of an n-channel in the transfer curve. Assuming the surface states are independent of nanotube density, the substrate-induced trap states per nanotube decrease with increasing nanotube coverage, as we have done here. This is further evidenced by the demonstration of ambipolar conduction shown in Figure S2. This was possible with the improvements in s-SWNT purifications that have allowed us to fabricate a denser network. As such, we expect the substrate effects to be minimized here, but not absent, since there is a significant coverage from the network. In addition, polymer wrapping and excess polymer may contribute to additional trap states.

## 4. Bandgap and Exciton Binding Energy

**Figure 5:**
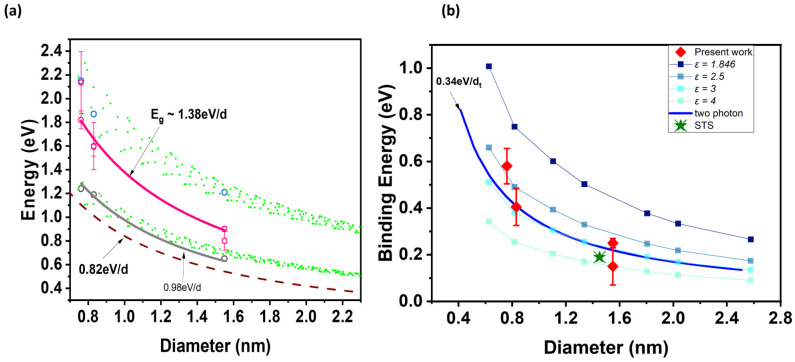
(**a**) An updated Kataura plot showing theoretical exciton transitions (closed green circles), measured E_11_ (open gray circles) and E_22_ (open blue circles) transitions from our s-SWCNT network *p-n* diodes (see Figure S3), and the tight binding bandgap (0.82 eV/d_t_) and measured bandgap values of our s-SWCNT network (open pink circles). We find a best fit of E_g_~1.38 eV/d_t_. (**b**) Binding energy dependence on diameter also showing data from other published works. See reference [22] for ε = 1.846, 2.5, 3 and 4. See reference [23] for the two photon data. See reference [2] for the STS data.

Next, we examine the extracted bandgap in the broader context of s-SWCNT studies. We show in Figure 5a the Kataura plot with updated values for the E_11_, E_22_, and E_g_ of our s-SWCNT networks. We fit our E_g_ and E_11_ values and derive the relation E_g_~1.38 eV/d_t_ and E_11_~0.98 eV/d_t_, respectively, where d_t_ is the nanotube diameter. The 1/d_t_ dependence comes from diameter quantum confinement and the linear dispersion relation of graphene.

We also show the binding energies calculated for our s-SWCNT networks and compare with previous theoretical [22] and experimental [2,23] works in Figure 5b. According to reference [22], the binding energy of the E_11_ excitons is related to the diameter, d_t_, through the function, $E_{b}=\frac{1}{d_{t}}(A+\frac{B}{d_{t}}+C\xi +D\xi^{2})$, where $\xi ={\left(-1\right)}^{v}.\cos\left(\frac{3\theta }{d_{t}}\right)$. Since we do not observe the chirality dependent effect, $v=\left(n-m\right)\;mod\;3$, in our experimental results, we use the relation $\xi =\cos\left(\frac{3\theta }{d_{t}}\right)$ for our comparison. $A=0.6724\;eVnm,B=-4.910\times 10^{-2}\;eV{nm}^{2},\;C=4.577\times 10^{-2}\;eV{nm}^{2},\;and\;D=-8.325\times 10^{-3}\;eV{nm}^{3}$ for nanotubes with a dielectric environment ε=1.846. For the binding energy dependence on dielectric environment, we adopt the scaling proposed by Perebeinos et al., $E_{b}\propto \varepsilon^{-1.4}$ [31]. We observe that the binding energy values of our network is best approximated with a dielectric environment ε ranging from 2.5–4. This range is consistent with the relatively low dielectric environment of our nanotubes. For example, this range is consistent with the two-photon excitation spectroscopy measurements of Dukovic et al. for s-SWCNT thin films embedded in a polymer matrix, where they derived the relation E_b_ = 0.34 eV/d_t_, consistent with binding energy values in a dielectric environment of ε=3 [22]. Also, Lin et al. used scanning tunneling spectroscopy (STS) to measure the binding energy of a semiconducting single nanotube at a height of ~3.5 nm from their metallic substrate, separated by bundles of arc discharge nanotubes [2].

Finally, we contrast our results to the single nanotube *p-n* diode studies where we observe ideal diode behavior and bandgap values that are below the E_11_ values. The small bandgap values measured in those studies arise from a significant doping-induced bandgap renormalization in the doped regions [26,32]. In this work, we measured the bandgap of largely undoped s-SWCNT networks located between the gated regions. This key difference is possible because coulombic screening lowers the doping per nanotube in the network compared to single nanotube devices, which reduces the amount of bandgap renormalization [33,34]. Therefore, with a larger bandgap in the doped regions of the network devices compared to the single nanotube devices, we are able to measure the electronic properties of the intrinsic region. Also, by focusing on diodes with *n*~2, we are guaranteed to measure the intrinsic bandgap, rather than the renormalized bandgap, however small the renormalization may be.

In conclusion, we measured the electronic bandgap of polymer-wrapped s-SWCNT networks by fabricating non-ideal diodes with diode ideality factor *n*~2. We show that the activation energy and excitonic levels follow a universal diameter dependence that allows the extraction of the bandgap and exciton binding energies. We analyzed our data in the context of bandgap renormalization due to dielectric screening. The techniques we demonstrated can also be applied to further the understanding of the electronic bandgaps and exciton binding energies of other nanoscale semiconductors like two-dimensional transition metal dichalcogenides [35,36,37].

## 5. Methods

### 5.1. Preparation of Semiconducting SWCNT Solutions

As discussed in more detail in our previous work [11], large-diameter semiconducting SWCNTs were sorted from raw arc discharge SWCNTs (Carbon Solutions Inc., Riverside, CA, USA). PFO-BPy (1 mg mL^−1^) obtained from American Dye Source was dissolved in 10 mL of toluene and mixed with the raw SWCNT in a 1:2 ratio. The solution was then sonicated using a horn tip sonicator in a cool water bath for 30 min with 1 s pulses (Branson digital sonifier, Division of Emerson, St. Louis, MO, USA) at 70% amplitude. Next, the sonicated solution was centrifuged at 15,000 rpm for 10 min (Hermle Z 36 HK centrifuge 221.22 V20 rotor, Sayreville, NJ, USA), and the semiconducting supernatant was collected and used as is.

The small diameter semiconducting SWCNTs were extracted from CoMoCAT SG65i material (CHASM, Boston, MA, USA) using PFO-BPy and PFO (purchased from American Dye Source, QC, Canada) for (6,5) and (7,5) SWCNTs, respectively. A quantity of 2 mg mL^−1^ of PFO-BPy or PFO was dissolved in toluene and used to disperse (6,5) and (7,5) SWCNTs, respectively, from 0.5 mg mL^−1^ of SG65i by tip sonication for 15 min at 40% intensity (Cole-Palmer CPX 750, 1⁄2” tip, Vernon Hills, IL, USA) in a cool bath of flowing water (~18 °C). Next, the tip-sonicated mixtures were immediately centrifuged at 20 °C and 13,200 rpm for 5 min (Beckman Coulter L-100 XP ultracentrifuge, SW-32 Ti rotor, Indianapolis, IN, USA) to remove the undispersed soot. The polymer-wrapped supernatants (PFO-BPy/(6,5) and PFO/(7,5)) were then centrifuged again at 20 °C and 24,100 rpm for 20 h to remove excess polymer. The pellet from each of the polymer-wrapped (6,5) and (7,5) solutions was then separated from the supernatant and redispersed in toluene. This process (pelleting and redispersion) was repeated until the absorption of the wrapping polymer (either PFO-BPy or PFO) approached that of the (6,5) or (7,5) S22 excitonic transition, after which the final pellet was then redispersed in toluene in a heated ultrasonic bath for more than an hour to yield nearly monochiral (6,5)/PFO-BPy or (7,5)/PFO s-SWCNT solutions.

### 5.2. Device Fabrication

To form a *p-n* diode on networks of SWCNTs, we fabricated the buried split gates in the 300 mm wafer fab of the College of Nanoscale Science and Engineering at SUNY Polytechnic Institute in Albany as described previously [11,25]. Briefly, the device was fabricated using standard lithography, deposition, and etch techniques in the SUNY Polytechnic Institute 300 mm fab. The fabrication started with a 300 mm poly-Si wafer, which was made highly conductive by phosphorous implantation with a concentration of ~10^19^ cm^−3^ to ~10^20^ cm^−3^, followed by annealing to activate the dopants, to form the back gate. A quantity of 100 nm of silicon dioxide (SiO_2_) dielectric was then deposited over the heavily p-doped silicon wafer using a wet thermal oxidation process. Next, the buried split gates were formed using 100 nm of polysilicon deposited over the dielectric and doped by ion implantation. Using standard photolithography and subtractive etch techniques, the polysilicon was etched to define the split gates with inter-gate spacing, G, ranging between 0.1 µm to 1 µm. On top of the split gates, 150 nm of SiO_2_ was further deposited using a plasma-enhanced chemical vapor deposition process (PECVD) and polished using chemical mechanical polishing (CMP) to achieve an atomically flat surface until a desired dielectric thickness of 100 nm was achieved. The CMP process was precisely controlled by checking the dielectric thickness at multiple intervals. To allow probes to land on the poly-Si bondpads for electrostatic gating, the dielectric above the bondpads was then subsequently etched. Onto these pre-fabricated structures, we then deposited the s-SWCNT networks using repetitive immersion and soaking in hot toluene at 120 °C for 10 min to remove excess polymer. This repetitive process was necessary to yield devices with n~2. To complete the device, we used electron beam lithography and oxygen plasma etching to define the s-SWCNT channel, L_ch_, and deposited 20 nm/20 nm of Ni/Au to form the source and drain contacts. See Figure S1 for a device schematic with a full description of dimensions.

### 5.3. Measurement Methods

All electrical measurements were performed in vacuum (<5 × 10^−5^ torr) at temperatures 300 K–340 K (for temperature dependent measurements, see Figure S3) using an Agilent B1500A (Santa Rosa, CA, USA) semiconductor parameter analyzer. Photocurrent measurements were carried out using an NKT photonics broadband laser dispersed through a monochromator.

### 5.4. Atomic Force Microscopy (AFM) Measurements

AFM measurements for SWCNT thickness were acquired using a Bruker Dimension icon AFM with ScanAsyst and gwyddion 2.6 software used for analysis. Scans were acquired across the SWCNT network with a scan rate of 0.2 Hz at 1024 × 1024 resolution using a ScanAsyst air–silicon tip. 

## Data Availability

Data will be made available upon request.

## Supplementary Materials

The following supporting information can be downloaded at: https://www.mdpi.com/article/10.3390/ma17153676/s1, Transfer curves of arc, (7,5), and (6,5) s-SWCNT network devices; normalized photocurrent spectra of arc, (7,5), and (6,5) s-SWCNT network diodes; fitted temperature dependent current–voltage (I-V) curves for arc, (7,5), and (6,5) diodes. References [38,39] are cited in the Supplementary Material.

## References

[1] Wang F., Dukovic G., Brus L.E., Heinz T.F. (2005). The Optical Resonances in Carbon Nanotubes Arise from Excitons. Science.

[2] Lin H., Lagoute J., Repain V., Chacon C., Girard Y., Lauret J.-S., Ducastelle F., Loiseau A., Rousset S. (2010). Many-body effects in electronic bandgaps of carbon nanotubes measured by scanning tunnelling spectroscopy. Nat. Mater..

[3] Jagannathan H., Anderson B., Sohn C.-W., Tsutsui G., Strane J., Xie R., Fan S., Kim K.-I., Song S., Sieg S. Vertical-Transport Nanosheet Technology for CMOS Scaling beyond Lateral-Transport Devices. Proceedings of the Technical Digest—International Electron Devices Meeting, IEDM.

[4] Petrosyants K.O., Silkin D.S., Popov D.A. (2022). Comparative Characterization of NWFET and FinFET Transistor Structures Using TCAD Modeling. Micromachines.

[5] Nish A., Hwang J.Y., Doig J., Nicholas R.J. (2007). Highly selective dispersion of single-walled carbon nanotubes using aromatic polymers. Nat. Nanotechnol..

[6] Mistry K.S., Larsen B.A., Blackburn J.L. (2013). High-yield dispersions of large-diameter semiconducting single-walled carbon nanotubes with tunable narrow chirality distributions. ACS Nano.

[7] Shulaker M.M., Hills G., Patil N., Wei H., Chen H.Y., Wong H.S., Mitra S. (2013). Carbon nanotube computer. Nature.

[8] Bishop M.D., Hills G., Srimani T., Lau C., Murphy D., Fuller S., Humes J., Ratkovich A., Nelson M., Shulaker M.M. (2020). Fabrication of carbon nanotube field-effect transistors in commercial silicon manufacturing facilities. Nat. Electron..

[9] Blackburn J.L. (2017). Semiconducting Single-Walled Carbon Nanotubes in Solar Energy Harvesting. ACS Energy Lett..

[10] Arnold M.S., Blackburn J.L., Crochet J.J., Doorn S.K., Duque J.G., Mohite A., Telg H. (2013). Recent developments in the photophysics of single-walled carbon nanotubes for their use as active and passive material elements in thin film photovoltaics. Phys. Chem. Chem. Phys..

[11] Oyibo G., Barrett T., Jois S., Blackburn J.L., Lee J.U. (2022). All-Carbon Nanotube Solar Cell Devices Mimic Photosynthesis. Nano Lett..

[12] Shea M.J., Arnold M.S. (2013). 1% solar cells derived from ultrathin carbon nanotube photoabsorbing films. Appl. Phys. Lett..

[13] Bindl D.J., Arnold M.S. (2013). Efficient Exciton Relaxation and Charge Generation in Nearly Monochiral (7,5) Carbon Nanotube/C 60 Thin-Film Photovoltaics. J. Phys. Chem. C.

[14] Avery A.D., Zhou B.H., Lee J., Lee E.-S., Miller E.M., Ihly R., Wesenberg D., Mistry K.S., Guillot S.L., Zink B.L. (2016). Tailored semiconducting carbon nanotube networks with enhanced thermoelectric properties. Nat. Energy.

[15] MacLeod B.A., Stanton N.J., Gould I.E., Wesenberg D., Ihly R., Owczarczyk Z.R., Fewox C.S., Folmar C.N., Hughes K.H., Zink B.L. (2017). Large n- and p-type thermoelectric power factors from doped semiconducting single-walled carbon nanotube thin films. Energy Environ. Sci..

[16] Zaumseil J. (2020). Recent Developments and Novel Applications of Thin Film, Light-Emitting Transistors. Adv. Funct. Mater..

[17] Zorn N.F., Berger F.J., Zaumseil J. (2021). Charge Transport in and Electroluminescence from sp3-Functionalized Carbon Nanotube Networks. ACS Nano.

[18] Zorn N.F., Zaumseil J. (2021). Charge transport in semiconducting carbon nanotube networks. Appl. Phys. Rev..

[19] Sze S. (2006). Physics of Semiconductor Devices.

[20] Fischer C.W. (1998). Elementary technique to measure the energy band gap and diffusion potential of pn junctions. Am. J. Phys..

[21] Rhiger D.R., Smith E.P., Kolasa B.P., Kim J.K., Klem J.F., Hawkins S.D. (2016). Analysis of III–V Superlattice nBn Device Characteristics. J. Electron. Mater..

[22] Capaz R.B., Spataru C.D., Ismail-Beigi S., Louie S.G. (2007). Excitons in carbon nanotubes: Diameter and chirality trends. Phys. Status Solidi B Basic Res..

[23] Dukovic G., Wang F., Song D., Sfeir M.Y., Heinz T.F., Brus L.E. (2005). Structural dependence of excitonic optical transitions and band-gap energies in carbon nanotubes. Nano Lett..

[24] Malapanis A., Perebeinos V., Sinha D.P., Comfort E., Lee J.U. (2013). Quantum efficiency and capture cross section of first and second excitonic transitions of single-walled carbon nanotubes measured through photoconductivity. Nano Lett..

[25] Dhakras P.A., Comfort E., Lee J.U. (2019). Ideal *p-n* Diodes from Single-Walled Carbon Nanotubes for Use in Solar Cells: Beating the Detailed Balance Limit of Efficiency. ACS Appl. Nano Mater..

[26] Malapanis A., Jones D.A., Comfort E., Lee J.U. (2011). Measuring carbon nanotube band gaps through leakage current and excitonic transitions of nanotube diodes. Nano Lett..

[27] Schießl S.P., Rother M., Lüttgens J., Zaumseil J. (2017). Extracting the field-effect mobilities of random semiconducting single-walled carbon nanotube networks: A critical comparison of methods. Appl. Phys. Lett..

[28] Lee J.U. (2007). Band-gap renormalization in carbon nanotubes: Origin of the ideal diode behavior in carbon nanotube *p-n* structures. Phys. Rev. B Condens. Matter Mater. Phys..

[29] Bonilla R.S., Wilshaw P.R. (2017). On the c-Si/SiO_2_ interface recombination parameters from photo-conductance decay measurements. J. Appl. Phys..

[30] Jones D.A., Lee J.U. (2011). Observation of the urbach tail in the effective density of states in carbon nanotubes. Nano Lett..

[31] Perebeinos V., Tersoff J., Avouris P. (2004). Scaling of excitons in carbon nanotubes. Phys. Rev. Lett..

[32] Comfort E., Lee J.U. (2016). Large Bandgap Shrinkage from Doping and Dielectric Interface in Semiconducting Carbon Nanotubes. Sci. Rep..

[33] Kshirsagar C., Li H., Kopley T.E., Banerjee K. (2008). Accurate Intrinsic Gate Capacitance Model for Carbon Nanotube-Array Based FETs Considering Screening Effect. IEEE Electron Device Lett..

[34] Aspitarte L., McCulley D.R., Bertoni A., Island J.O., Ostermann M., Rontani M., Steele G.A., Minot E.D. (2017). Giant modulation of the electronic band gap of carbon nanotubes by dielectric screening. Sci. Rep..

[35] Ross J.S., Klement P., Jones A.M., Ghimire N.J., Yan J., Mandrus D.G., Taniguchi T., Watanabe K., Kitamura K., Yao W. (2014). Electrically tunable excitonic light-emitting diodes based on monolayer WSe_2_ p–n junctions. Nat. Nanotechnol..

[36] Pospischil A., Furchi M.M., Mueller T. (2014). Solar-energy conversion and light emission in an atomic monolayer *p–n* diode. Nat. Nanotechnol..

[37] Baugher B.W.H., Churchill H.O.H., Yang Y., Jarillo-Herrero P. (2014). Optoelectronic devices based on electrically tunable *p–n* diodes in a monolayer dichalcogenide. Nat. Nanotechnol..

[38] Rother M., Zakharko Y., Gannott F., Zaumseil J. (2016). Understanding Charge Transport in Mixed Networks of Semiconducting Carbon Nanotubes. ACS Appl. Mater. Interfaces.

[39] Brohmann M., Berger F.J., Matthiesen M., Schießl S.P., Schneider S., Zaumseil J. (2019). Charge Transport in Mixed Semiconducting Carbon Nanotube Networks with Tailored Mixing Ratios. ACS Nano.

