# Supplementary material for: Measuring the Electronic Bandgap of Carbon Nanotube Networks in Non-Ideal p-n Diodes
Source: Materials (Basel). 2024 Jul 25;17(15):3676. doi: 10.3390/ma17153676 (PMC11312849; doi:10.3390/ma17153676)
Supplement: Supplementary file 1 [file materials-17-03676-s001.zip › materials-3091835-supplementary.pdf]

Measuring the Electronic Bandgap of Carbon Nanotube Networks in Non-Ideal *p-n* Diodes

Gideon Oyibo [1], Thomas Barrett [1], Sharadh Jois [1], Jeffrey L. Blackburn [2] and Ji Ung Lee [1,*]

[1] College of Nanotechnology, Science, and Engineering, State University of New York-Albany, Albany, NY 12203, USA; oyibog@sunypoly.edu (G.O.); barrettl@sunypoly.edu (T.B.); joiss@sunypoly.edu (S.J.)
[2] National Renewable Energy Laboratory, Golden, CO 80401, USA; jeffrey.blackburn@nrel.gov
* Correspondence: jlee1@albany.edu

Supplementary Information

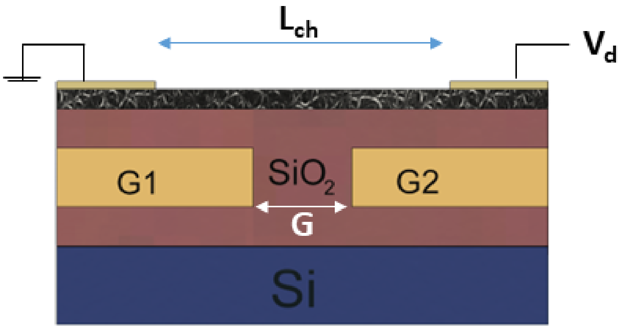

Figure S1: Device schematic showing the channel length between the contact electrodes, L_{ch} and the spacing between the split gates, G. L_{ch} for all devices in this work is 3um and G spacing ranges from 0.1um to 1um. The thickness of the dielectric between the split gate and the back gate is 100nm, the thickness of each split gate is 100nm and the thickness of the dielectric above the split gate is 100nm.

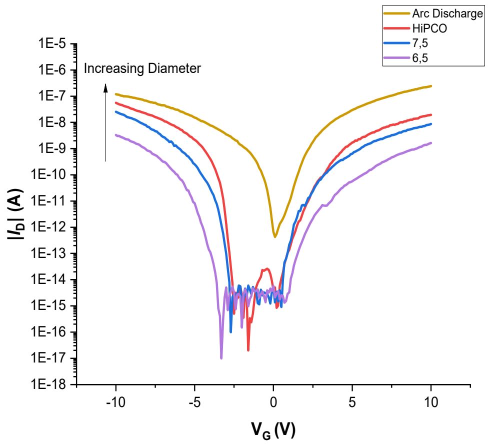

Figure S2: Representative transfer curves for devices from Arc, HiPCO, (7,5) and (6,5) nanotubes. The gate voltage is swept using the back gate V_{BG} as shown in Fig. 1a with a drain voltage V_D of -0.1V. Channel length between source and drain is 3um. Transfer curves are taken while the split gates are floating.

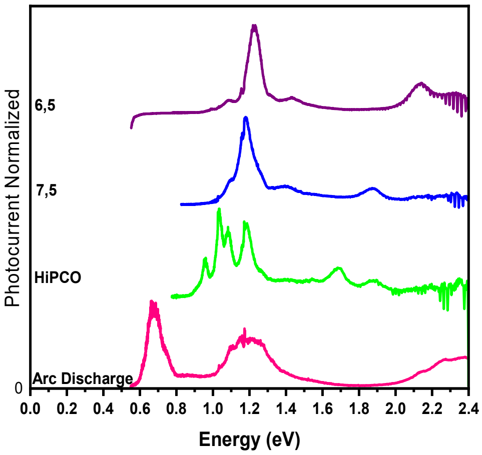

Figure S3: Measured photocurrent spectra from our s-SWNT network p-n diodes.

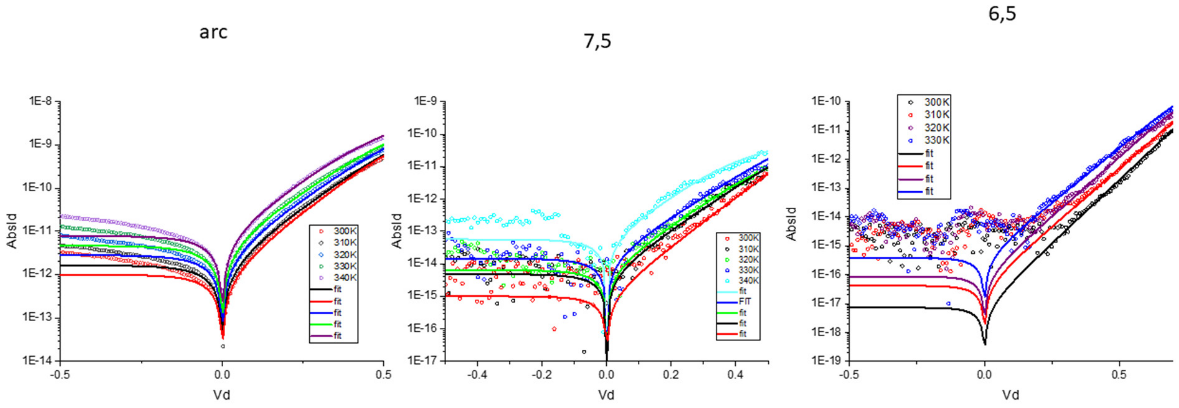

Figure S4: Representative temperature dependent current voltage (I-V) measurements for arc, (7,5) and (6,5) devices.

HiPCO Device Results in Comparison to the Monochiral s-SWCNTs

Within the network s-SWCNT diodes we have examined, a sharp contrast is seen when we include the results from the polychiral HiPCO s-SWCNT devices (please see Figs. S5-S6 below where we include the HiPCO device results along with the data from the manuscript).

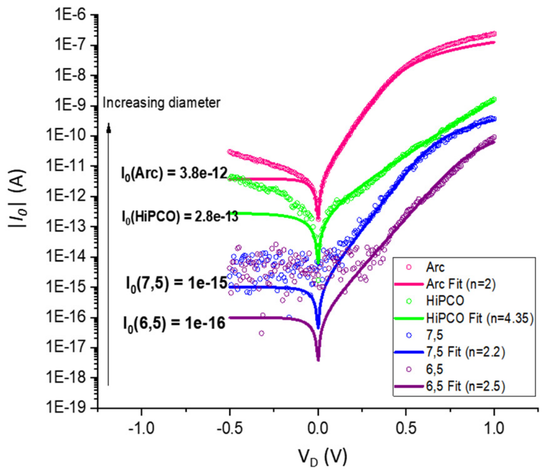

Figure S5: Representative diode IV curves including the HiPCO network. Note, the HiPCO device has n>4 (compare to Fig 2a), much higher than the other s-SWCNTs.

As one can see in Fig. S5, the HiPCO diode is highly nonideal compared to the monochiral diodes. The HiPCO p-n diode has an ideality factor n>4. Whereas diodes with n~1 and n~2 are well established, no single theory can explain diodes with n>4. The highly non-ideal nature of the HiPCO devices is not due to defects based on our Raman results but rather due to the multiple bandgaps present in the network, as shown in the photocurrent spectra (Fig. S2). We speculate that the large variation in the bandgap within the network effectively increases the density of trap states, which in turn increases the generation and recombination processes beyond what is already present in the network of the monochiral s-SWCNTs.

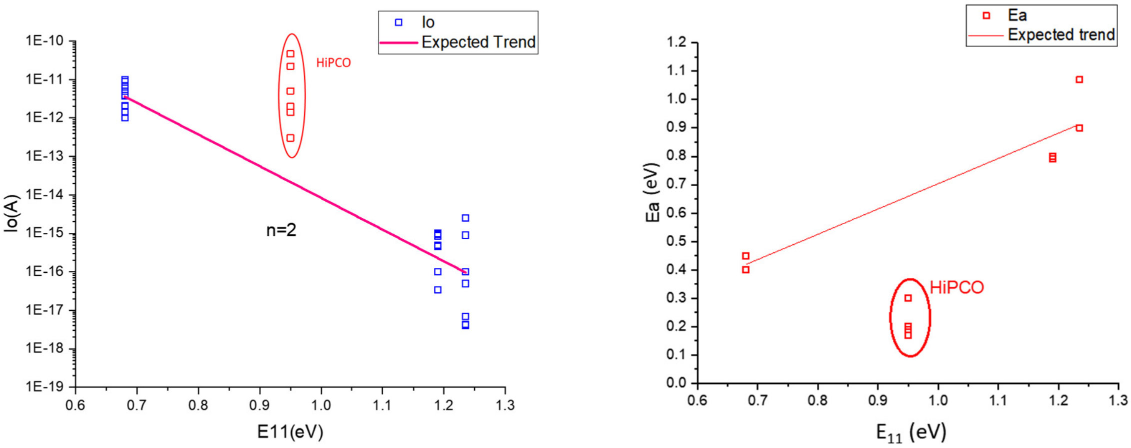

Figure S6: ***Results including HiPCO s-SWCNTs:*** *(Left) Leakage currents of different network s-SWCNTs. Note, the HiPCO s-SWCNT diodes have leakage current, I_0 which deviates from the monochiral s-SWCNTs that we reported in the manuscript (compare to Fig 2b). The line is a fit to the data using only the monochiral s-SWCNTs. (Right) The activation energy Ea vs E11 (Eg=2xEa for the data in Fig. 3b for n=2 diodes). The Ea for HiPCO s-SWCNTs doesn't match the general trend seen for the monochiral s-SWCNT devices.*

In looking at the transfer curves (Fig. S1), one would have guessed that HiPCO s-SWCNTs should behave similarly to the large bandgap 6,5 and 7,5 s-SWCNTs. Although this behavior is similar to other studies where the smallest bandgap s-SWCNT in the network dominates the electron and hole transport, (see references 38, 39), the transfer curve does not give an accurate picture of the generation and recombination mechanisms on the network. To further understand this, we look at the behavior of the diodes formed on the s-SWCNT networks as shown in Fig. S6 (Left), which is in complete contrast to what is expected from the transfer curves. In fact, despite having larger bandgap s-SWCNTs than the Arc Discharge s-SWCNTs, the HiPCO diodes have leakage currents comparable to the Arc Discharge devices.

In light of the HiPCO results, the trends in the monochiral s-SWCNTs become clear (Fig. 2 and Fig. S6 – right). Even with the large scatter in the data in Fig. 2 (Io vs E11), they point to a trend consistent with the bandgap of largely monochiral s-SWCNTs. The line in Fig. 2 is a fit to the scattered data of the monochiral s-SWCNTs and results in a slope with an ideality factor 2, as discussed in the main text. Similarly, the trend in Fig. S6-left of Ea vs E11 matches the expected trend once the HiPCO results are excluded.
